# Supplementary material for: Effect of mode of healthcare delivery on stress and intention to quit among physicians in Canada during the COVID-19 pandemic
Source: PLOS Glob Public Health. 2024 Jul 18;4(7):e0003377. doi: 10.1371/journal.pgph.0003377 (PMC11257304; doi:10.1371/journal.pgph.0003377)
Supplement: Attachment — Submitted filename: PGPH-D-24-00506 - Response to reviewers.docx [file pgph.0003377.s001.docx]

PGPH-D-24-00506
Effect of mode of healthcare delivery on stress and intention to quit among physicians in Canada during the COVID-19 pandemic
PLOS Global Public Health

Response to Reviewers

| **Reviewer 1 comments** | **Authors response** |
| --- | --- |
| 1. As the authors mentioned in the limitation section, the cross-sectional nature of the study meant it was not possible to infer causation. Some of the remarks may need to be more cautious; e.g. in the abstract, it states online or blended healthcare delivery decreased the odds of intention to quit, while it may be more accurate to say it was associated with a decreased odds. | Thank you for the comment. The wording has been revised throughout the manuscript. |
| 2. In the last paragraph of the Introduction, the Poland study showed that high levels of stress and fear was associated with job satisfaction - presumably this was 'negatively' associated | Correct, this has been added. Thank you. |
| 3. Response rate was 54.9% and data was collected end of 2021 (2 years after the pandemic started). It is unclear whether physicians who really wanted to quit have already done so earlier before the survey was done, and whether response bias could have played a role - those who wanted to quit were less likely to complete the survey. This may need to be clarified or included as a limitation. | Thank you, this has been added to the limitation section of the revised manuscript (under selection bias). |
| 4. In the results section, 20.8% expressed intention to quit their job, while 7.5% were due to stress, burnout or mental health concerns. According to the table, 11.1% were retiring anyway, which explains half of the people intending to quit. This may also explain why more physicians age >55 years were more likely to have this intention, or perhaps not so interested to adjust to online / blended healthcare delivery. What do the author's think about this? | Thank you for the question. Percentages presented in Table 1 are based on the total sample. These percentages do not add up to 100% because participants were asked to choose all answers that may apply. Out of the weighted sample of 195,709 with intention to quit current job within 2 years, 2,295 (11.7%) reported both "retiring" and "stress or burnout or mental health concerns" as reasons for their intention to quit. This information has been added to the Discussion section of the revised manuscript. |
| 5. The authors in the discussion section stated that providing both in-person and online care may reduce concerns from physicians re: providing care to patients. What are the implications to practice now in the endemic phase of COVID-19? | Thanks for your note. This has been added to the discussion section of the revised manuscript. |
| 6. IPC formally trained were likely to quit their job. The authors suggested that this may be due to pressure to adhere to safety protocols. In my opinion, the physicians would want to adhere to the safety protocols given IPC measures are risk mitigation measures to reduce infection risk. However, the need for formal training in IPC suggests that these were high risk and more stressful areas to work in, so there may be other confounding factors for this finding. | Thank you. After careful consideration of the findings, we completely agree with the review and have modified the interpretation accordingly. |
| There are a few areas to clarify, especially in the discussion section. I look forward to hearing the feedback and responses from the authors to clarify these issues. | Thank you for your feedback and comments. They have contributed to the improvement of the manuscript. |
| **Reviewer 2 comments** | **Authors response** |
| Did the survey look at the specialty of the responders? Studies have shown that physicians in different specialties or sub-specialties encounter different levels of stress during the pandemic. For example, a emergency surgeon's level of stress would be expected to be higher than a psychiatrist. | Thank you for the comment. Unfortunately, this information was not available. This has been added as a potential limitation in the revised manuscript. |
| The survey was conducted in 2021, was this during the height of the pandemic restrictions in Canada? Does this correspond to the movement restrictions or period of lock down? | During that period, 80% of eligible Canadians were fully vaccinated. However, the Delta variant caused a resurgence in cases, leading to the reintroduction of some restrictions, especially for unvaccinated individuals in indoor settings. This has been added to the methods of the revised manuscript. |
|  | Thank you for your feedback and comments. They have contributed to the improvement of the manuscript. |
